# Supplementary material for: The first North American Propterodon (Hyaenodonta: Hyaenodontidae), a new species from the late Uintan of Utah
Source: PeerJ. 2019 Nov 22;7:e8136. doi: 10.7717/peerj.8136 (PMC6876642; doi:10.7717/peerj.8136)
Supplement: Supplemental Information 1 [file peerj-07-8136-s001.docx]

SUPPLEMENTARY DATA

**The first North American *Propterodon* (Hyaenodonta: Hyaenodontidae), a new species of from the late Uintan of Utah**

Shawn P. Zack^1,2^

^1^ Department of Basic Medical Sciences, University of Arizona College of Medicine-Phoenix, Phoenix, Arizona, U.S.A.

^2^ School of Human Evolution and Social Change, Arizona State University, Tempe, Arizona, U.S.A.

**Part A. Character List**

Codes following most characters refer to character numbers in either Rana et al. (2015) (R) or Borths and Stevens (2019a) (BS).

1. Premolar diastemata (R3)

(0) premolar series crowded, no diastema between p2 and p3, gap between c and p2 small

(1) small diastema between p2 and p3, gap between c and p2 small

(2) large diastema between p2 and p3, gap between c and p2 small

(3) large diastema between p2 and p3, gap between c and p2 large

(4) no diastema between p2 and p3, large gap between c and p2

2. Enamel (R4)

(0) enamel thin and smooth

(1) enamel crenulated

3. p1 presence (R5)

(0) present

(1) absent

4. p1 roots (R6)

(0) two-rooted

(1) single-rooted

5. p2 talonid development (R7)

(0) talonid short

(1) talonid elongated and cutting

6. Relative heights of p2 and p3 (R8)

(0) p2 lower than p3

(1) p2 as high as p3

(2) p2 higher than p3

7. p3 talonid development (R9)

(0) talonid short

(1) talonid long

8. p3 entoconid presence (R10)

(0) entoconid absent

(1) entoconid present

9. Relative lengths of p3 and p4 (R11)

(0) p3 shorter than p4

(1) p3 longer than p4

10. p4 paraconid development (R12)

(0) paraconid small or absent

(1) paraconid well-developed

11. p4 metaconid development (R13)

(0) metaconid absent

(1) metaconid present but weakly developed (= metaconid ridge)

12. p4 buccal talonid cusps (R14)

(0) only one well-developed cusp present

(1) two subequal cusps present

13. p4 entoconid presence (R15)

(0) absence of an entoconid

(1) presence of an entoconid

14. p4 accessory cusp presence (R16)

(0) absence of an accessory cusp

(1) presence of an accessory cusp

15. p4 hypoconid development (R17)

(0) hypoconid low

(1) hypoconid high and secant

16. p4 talonid basin development (R18)

(0) talonid narrow and shallow

(1) talonid wide and deep postfossid

17. p4 cingulid development (R19)

(0) absence of cingulid

(1) presence of precingulid and postcingulid

18. p4 inflation (R20)

(0) compressed transversely

(1) inflated transversely, only on the talonid part

(2) inflated transversely in mesial and distal part

19. p4 protoconid proportions (R21, modified)

(0) p4 protoconid very low

(1) p4 protoconid taller, but height less than p4 length

(2) p4 height greater than length

20. p4 height

(0) small, protoconid lower than m1-2 protoconids

(1) taller, protoconid taller than m1 but lower than m2 protoconid

(2) tall, protoconid taller than m1-2 protoconids

21. Lower molars (R22)

(0) paraconid poorly mesially located

(1) paraconid mesially located

(2) paraconid very mesially located

22. m2 metaconid position (R23)

(0) disto-lingual to the protoconid

(1) directly lingual to the protoconid

(2) mesio-lingual to the protoconid

23. Development of m2 metaconid (R24)

(0) metaconid taller than paraconid

(1) metaconid subequal to paraconid height

(2) metaconid lower than paraconid

(3) metaconid absent

24. Lower molars (R25)

(0) paraconid small or enlarged

(1) paraconid strongly reduced

25. m1 and m2 entoconid development (R26)

(0) entoconid or entocristid as tall as hypoconid

(1) entoconid or entocristid present but lower than hypoconid

(2) entoconid and entocristid strongly reduced or absent, talonid trenchant

26. m1 and m2 entoconid shape (R27, modified)

(0) well-developed and bulbous

(1) crestiform but clearly distinct from hypoconulid

(2) crestiform, part of a continuous crest from hypoconulid apex

27. m1-2 cristid obliqua orientation

(0) oblique

(1) more longitudinal

28. m1-2 hypoconulid position

(0) relatively buccal, close to hypoconid, hypocristid short

(1) relatively lingual, distant from hypoconid, hypocristid elongate

29. m1 and m2 talonid proportions (R29, modified)

(0) talonid narrow relative to length

(1) talonid broad relative to length

30. m1 and m2 talonid width (R31, modified)

(0) talonid narrow relative to trigonid

(1) talonid wide relative to trigonid

31. Relative sizes of m1 and m2 (R32)

(0) m1 subequal or longer than the m2

(1) m1 shorter than the m2

32. m3 size (R33, modified)

(0) m3 trigonid much larger than m2 trigonid

(1) m3 subequal or slightly longer or smaller than the m2

(2) m3 distinctly smaller than the m2

(3) m3 absent

33. Lower molar trigonid heights (R34)

(0) trigonid high

(1) trigonid low on all molars

(2) trigonid low on m1 and m2

34. Lower molar cingulid development (R36)

(0) buccal cingulids very weak or absent on lower molars

(1) ectocingulid well-developed but postcingulid absent

(2) postcingulid well-developed but ectocingulid absent

(3) ecto- and postcingulids well-developed but separate

(4) ecto- and postcingulids well developed and continuous to form a complete buccal cingulid

35. Lower molar buccal enamel distention (R37)

(0) very strong

(1) moderately developed

(2) reduced

36. Number of upper incisors (BS71, modified)

(0) three

(1) two or fewer

37. P3 protocone development (R38, modified)

(0) lobe of the protocone absent

(1) lobe of the protocone present but small

(2) protocone well developed and individualized

38. P4 parastyle development (R40)

(0) parastyle very reduced to absent

(1) parastyle present, but small

(2) parastyle strong

39. P4 protocone position (R41)

(0) protocone aligned transversely with the paracone

(1) protocone shifted mesially versus paracone

40. Separation of P4 protocone from paracone (R42)

(0) protocone bulbous and separated from the paracone

(1) protocone poorly separated from the paracone

41. P4 lingual accessory cusps (R43)

(0) absence of accessory cusps in the protocone area

(1) presence of accessory cusps in the protocone area

42. P4 metastyle development (R44)

(0) short metastyle

(1) elongated metastyle

43. M1 and M2 postmetacrista (R45, modified)

(0) postmetacrista straight with carnassial notch between metacone and metastyle portions

(1) postmetacrista arcuate without carnassial notch

44. M1 and M2 metastyle length (R46)

(0) metastyle short (the carnassial blade shorter than the parametacrista)

(1) metastyle medium

(2) metastyle elongated (the carnassial blade longer than one and a half length of the parametacrista)

45. M1 parastyle lobe development (R47)

(0) parastyle lobe prominent and buccally directed

(1) parastyle lobe prominent and mesially directed

(2) parastyle lobe reduced and mesially directed

46. M2 parastyle lobe development (R48)

(0) parastyle lobe prominent and buccally directed, with a distinct stylocone as well as paracone

(1) parastylar lobe reduced, stylocone absent or poorly developed

47. M1 and M2 buccal cusp height (R49, modified)

(0) paracone higher than metacone

(1) paracone as high as metacone

(2) paracone lower than metacone

48. M1 and M2 buccal cusp fusion (R50)

(0) paracone and metacone separate almost at basis

(1) paracone and metacone fused up to mid-height

(2) paracone and metacone almost or completely fused

49. M1 and M2 size (R51)

(0) M1 subequal or larger than the M2

(1) M1 smaller than the M2

50. M1 and M2 postparaconule crista (R52)

(0) postparaconule crista present

(1) postparaconule crista absent

51. M1 and M2 premetaconule crista (R53)

(0) premetaconule crista present

(1) premetaconule crista absent

52. M1-2 metacingulum length (R54)

(0) metacingulum long, extends along base of metastyle

(1) metacingulum short, terminating at base of metacone

53. M1-2 metaconule (R55)

(0) present

(1) absent as a definable cusp

54. M1 and M2 mesial and distal cingular development (R56, modified)

(0) mesial and distal cingulum absent on M1 and M2

(1) cingula present but separated at least on M1

(2) cingula present and joined lingually at least on M1

55. M1 and M2 protocone position (R58)

(0) protocone centered transversely with respect to the position of the paracone and the metacone

(1) protocone markedly shifted mesially at least at paracone level

56. Separation of M1-2 protocones from buccal cusps (R60)

(0) M1-2 protocones well lingual to buccal cusps

(1) M1-2 protocones closely appressed to buccal cusps

57. M3 metacone (R61)

(0) metacone present and lower than the paracone

(1) metacone absent

58. M3 size (R62)

(0) M3 subequal to the M1 and M2

(1) M3 slightly smaller than M1 and M2

(2) M3 much smaller than M1 and M2

(3) M3 absent

59. Prefossa/postfossid shearing (R63)

(0) absent

(1) present

60. First mental foramen (R1)

(0) below p1

(1) below p2

61. Second mental foramen (R2)

(0) below p3

(1) below p4

62. Genioglossus attachment and symphysis orientation (R80)

(0) at the base of a horizontal mandibular symphysis

(1) halfway up a vertical symphysis

63. Mandible inflection anterior to angular process (BS56)

(0) present

(1) absent

64. Angular process morphology (BS57)

(0) distinct process with medial inflection

(1) gently curved process in line with mandibular corpus

(2) ventral inflection

65. Mandibular condyle position (BS58)

(0) superior to m3 alveolus

(1) directly distal to m3 alveolus

(2) inferior to m3 alveolus

66. Coronoid process shape (BS59)

(0) tall, anterior and posterior slopes similar

(1) tall, posterior slope concave

(2) low, rounded

67. Anterior coronoid angle relative to horizontal ramus (BS60)

(0) near vertical, 90 to 100 degrees

(1) slight posterior inclination, 100 to 110 degrees

(2) strong posterior inclination, greater than 110 degrees

68. Masseteric fossa depth (BS61)

(0) deeply excavated with strong anterior angle, inferior margin well-defined

(1) rounded anterior margin, little inferior definition

(2) deep fossa but poorly defined inferior margin

69. Nuchal crest morphology (BS102)

(0) medial to lateral trend from apex to mastoid

(1) dorsolateral margin tapers medially with thin connection to exoccipital

(2) lateral margins trend medially, very weak ridge connects to exoccipital

70. Facial wing of the lacrimal (BS103)

(0) extensive (larger than orbit diameter)

(1) moderate (slightly longer than orbit diameter)

(2) reduced (shorter than orbit diameter)

71. Foramen rotundum size (BS104)

(0) slightly larger than foramen ovale

(1) much larger than foramen ovale

72. Palatal rugosity or torus at distal margin of palate (BS105)

(0) well-expressed

(1) smooth

73. Zygomatic arch contact (BS106)

(0) short contact between zygomatic and squamosal

(1) extensive contact between zygomatic and squamosal

74. Superior squamosal morphology (BS107)

(0) superior and inferior margins parallel

(1) torsion along superior margin

75. Foramen ovale orientation (BS108)

(0) anterior orientation

(1) palatal orientation

76. Exoccipital condyle position (BS109)

(0) tall, lateral placement around foramen magnum

(1) ventral placement around foramen magnum

77. Notch between occipital condyles (BS110)

(0) ring-like with no rostral excavation

(1) rounded indentation with condyles meeting medially below foramen magnum

(2) deep excavation with occipital "processes" following notch

78. Postglenoid process (BS111, modified)

(0) vertical orientation

(1) strong anterior curvature

79. Posterior orbital process (BS112)

(0) present, strong expression

(1) present, weak with frontal "peaked"

(2) absent

80. Frontal furrow (BS113)

(0) absent or indistinct

(1) present and well-defined

81. Palatine and pterygoid medial contact (BS114)

(0) parallel posterior to palatine torus

(1) palatines trend medially or partially fuse

(2) fused entire extent of palatines

82. Pterygoid size (BS115)

(0) broad, ventral projection anteriorly extensive

(1) short anterior extent, trends medially

83. Frontoparietal suture in dorsal view (BS116)

(0) steep constriction

(1) gentle curvature

84. Lateral expansion of the mastoid process (BS117)

(0) projects to midpoint of mandibular fossa

(1) projects beyond mandibular fossa

85. Mastoid/paroccipital process (BS118)

(0) short tubercle

(1) well-defined, prong-like process

86. Process at maxilla/jugal suture (BS119)

(0) present

(1) absent

87. Squamosal constriction in dorsal view (BS120)

(0) present, squamosal does not extend laterally

(1) absent, squamosal expanded laterally

88. Squamosal ventral projection (BS121)

(0) same transverse plane as petrosal

(1) ventral to petrosal

89. Posterior braincase (BS122)

(0) broad lateral expansion

(1) narrow

90. Subarcuate fossa morphology (BS123)

(0) cup-shaped (see *Pterodon*)

(1) shallow and horseshoe-shaped (see *Hyaenodon*)

91. Bridge of the stylomastoid foramen primitivum (BS124)

(0) absent or slender

(1) robust

(2) roofed over with secondary stylomastoid foramen

92. Mastoid sinus lateral to foramen stylomastoid primitivum (BS125)

(0) absent

(1) present

93. Ridge of bone dividing posterior petrosal sinus from foramen stylomastoid primitivum (BS126)

(0) present

(1) reduced to absent

94. Posterior petrosal sinus (BS127)

(0) absent

(1) small

(2) greatly inflated

95. Humerus cross-section above brachial flange (BS128)

(0) triangular

(1) rounded

96. Brachial flange expression (BS129)

(0) medium

(1) enlarged

(2) reduced

97. Entepicondylar foramen (BS130)

(0) present, rounded

(1) present, elongate

(2) absent

98. Medial epicondyle (BS131)

(0) large (bulbous)

(1) reduced (elongate)

99. Capitulum morphology (BS132)

(0) rounded, clearly separated

(1) cylindrical

100. Greater tubercle of the humerus (BS133)

(0) prominent, taller than humeral head

(1) subequal to height of humeral head

101. Proximal trochlear notch orientation (BS134)

(0) lateral position on shaft

(1) medial position on shaft

102. Radial notch orientation (BS135)

(0) curved and faces laterally

(1) flattened and faces anteriorly

103. Olecranon process length (BS136)

(0) longer than trochlear notch

(1) subequal or shorter than trochlear notch

104. Olecranon process orientation (BS137)

(0) projects medially

(1) projects ventrally

105. Third trochanter of femur (BS138)

(0) large

(1) small

106. Astragalar foramen (BS139)

(0) large

(1) reduced

107. Astragalar condyles divided by (BS140)

(0) shallow depression

(1) well-defined fossa or groove

108. Astragalar condyle orientation (BS141)

(0) oblique relative to astragalar neck

(1) parallel to astragalar neck

109. Astragalar head relative to condyles (BS142)

(0) horizontal orientation

(1) slight vertical orientation

110. Sustentacular facet connection to astragalar head (BS143)

(0) clearly separated from astragalar head

(1) grades into articulation of astragalar head

111. Sustentacular facet position on astragalar neck (BS144)

(0) plantar astragalar neck

(1) medial astragalar neck

112. Peroneal tubercle (BS145)

(0) distinct and separated from cuboid facet

(1) part of a flange that grades to cuboid facet

113. Cuboid facet inclination (BS146)

(0) perpendicular to calcaneal neck

(1) plantar inclination

114. Calcaneal neck trend (BS147)

(0) dorsal and plantar margins parallel

(1) tapers proximally to calcaneal tuberosity

115. Astragalar facet angle (BS148)

(0) oblique orientation to calcaneal neck

(1) parallels calcaneal neck

**Part B. List of Materials Used to Construct the Character Taxon Matrix**

**Institutional abbreviations** (excluding those given in the main text)—**BMNH**, Natural History Museum, London, United Kingdom; **CGM**, Cairo Geological Museum, Cairo, Egypt; **DPC**, Duke University Primate Center (now Duke University Lemur Center), Durham, North Carolina, U.S.A.;; **F:AM**, Frick Collection, American Museum of Natural History, New York, USA; **GMH**, Geiseltalmuseum der Martin Luther-Universität, Halle, Germany; **GU/RSR/VAS**, Department of Geology, H.N.B. Garhwal University, Srinagar, Uttarakhand, India; **MNHN.F.QU**, Muséum National d’Histoire Naturelle, Quercy Collection, Paris, France; **NMMP-KU**, National Museum of Myanmar, Kyoto University, Yangon, Myanmar; **NSM**, National Science Museum, Tokyo, Japan; **PIN**, Institute of Paleontology, Academy of Sciences, Moscow, Russia; **UCMP**, University of California, Museum of Paleontology, Berkeley, California, USA; **UM**, Museum of Paleontology, University of Michigan, Ann Arbor, Michigan, USA; **USGS**, United States Geological Survey, Denver registry, Denver, Colorado, USA (transferred to USNM); **USNM**, Department of Paleobiology, United States National Museum of Natural History, Smithsonian Institution, Washington D.C., USA; **UW**, University of Wyoming Geological Museum, University of Wyoming, Laramie, Wyoming, USA; **WIF/A**, Wadia Institute fossil collection, Wadia Institute of Himalayan Geology, Dehradun, India; **YPM**, Yale Peabody Museum, Yale University, New Haven, Connecticut, USA; **YPM-PU**, Princeton University collection, Yale Peabody Museum, Yale University, New Haven, Connecticut, USA.

*Maelestes gobiensis*: Wible et al. (2009)

*Altacreodus magnus*: Lillegraven (1969)

*Oxyaenoides bicuspidens*: GMH XIV-2848; Crochet et al. (1976); Lange-Badré and Haubold (1990); Morlo and Habersetzer (1999)

*Eurotherium* spp.: Polly (1996)

*E. matthesi*: GMH XIV-3419; Lange-Badré and Haubold (1990)

*E. theriodis*: Van Valen (1965)

*Prodissopsalis eocaenicus*: GMH VI-91; Lange-Badré and Haubold (1990)

*Leonhardtina gracilis*: Lange-Badré and Haubold (1990)

*Proviverra typica*: UCMP 140641, 140642, 140643; Van Valen (1965); Lange-Badré and Haubold (1990); Polly (1996)

*Allopterodon* spp.

*A. bulbosus*: Lange-Badré (1979); Lange-Badré and Mathis (1992)

*A. minor*: Lange-Badré (1979); Lange-Badré and Mathis (1992)

*Cynohyaenodon cayluxi*: AMNH 11050, 11052, 11054, 11055, 11056; Lange-Badré (1979); Lange-Badré and Mathis (1992)

*Paracynohyaenodon* spp.

*P. magnus*: Crochet (1988, 1991); Lange-Badré and Mathis (1992)

*P. schlosseri*: Lange-Badré (1979); Crochet (1991)

*Quercytherium* spp.

*Q. simplicidens*: MNHN.F.QU 8642; Lange-Badré (1979); Crochet (1991)

*Q. tenebrosum*: Lange-Badré (1979); Crochet (1991)

*Morlodon vellerei*: Solé (2013)

*Matthodon menui*: Solé et al. (2014a)

*Matthodon tritens*: Lange-Badré and Haubold (1990); Morlo and Habersetzer (1999)

*Boritia duffaudi*: Solé et al. (2014a)

*Preregidens langebadrae*: Solé et al. (2015)

*Indohyaenodon raoi*: GU/RSR/VAS 273, 321, 652, 740, 767, 807, 1630, 1631, 1680, 1721; Bajpai et al. (2009); Rana et al. (2015)

*Paratritemnodon indicus*: WIF/A 1102, 1103; Ranga Rao (1973); Kumar (1992)

*Kyawdawia lupina*: NMMP-KU 0042, 1161; Egi et al. (2005); Peigné et al. (2007); Bonis et al. (2018)

*Glibzegdouia tabelbalaensis*: Solé et al. (2014b)

*Masrasector* spp.

*M. aegypticum*: CGM 30978, YPM 20944, 30019, 30020, 30030

*M. ligabuei*: Crochet et al. (1990); Holroyd (1994)

*M. nananubis*: DPC 9274, 10358, 11990B, 12157, 15211; Borths and Seiffert (2017)

*Teratodon* spp.

*T. enigma*: Savage (1965)

*T. spekei*: Savage (1965)

*Brychotherium elphamos*: DPC 11569, 11990, 17627, 20243; Borths et al. (2016)

*Anasinopa leakeyi*: BMNH M.19081; Savage (1965)

*Dissopsalis* spp.

*D. carnifex*: AMNH FM 19401, 19402, 93027; Colbert (1933); Barry (1988)

*D*. *pyroclasticus*: Savage (1965); Barry (1988); Werdelin (2019)

*Furodon crocheti*: Solé et al. (2014b, 2016)

*Pterodon dasyuroides*: NSM DBR-61; Lange-Badré (1979); Polly (1996)

*Akhnatenavus* spp.

*A. leptognathus*: Osborn (1909); Holroyd (1999)

*A. nefertiticyon*: DPC 15653, 18242; Borths et al. (2016)

*Hyainailouros/Megistotherium* spp.: Polly (1996)

*H. napakensis*: Savage (1965)

*H. sulzeri*: Ginsburg (1980); Morales et al. (1998); Borths and Stevens (2019b)

*M. osteothlastes*: Savage (1973); Rasmussen et al. (1989)

*Apterodon macrognathus*: AMNH FM 13236, 13237, 13248, 92794, YPM 18127, 20945, 30035, 33221; Andrews (1906); Osborn (1909); Szalay (1967); Polly (1993)

*Galecyon peregrinus*: AMNH FM 56320, USNM 509676, UW 9864, YPM 32230

*Galecyon chronius*: USGS 9276, 10284, 10374, 15956, 25399, USNM 487920, 511004, 521860, YPM 23341

*Prototomus minimus*: Smith and Smith (2001); Solé et al. (2013)

*Prototomus phobos*: UM 68075, 73134, YPM-PU 13019; Gingerich and Deutsch (1989)

*Prototomus secundarius*: USGS 6666, 9066, 9095, USNM 495126; Gingerich and Deutsch (1989)

*Gazinocyon whitiae*: AMNH FM 4780, 15606, USNM 19347, 510864, 510979, YPM 29839/29585

*Sinopa* spp.: Polly (1996)

*S. grangeri*: AMNH FM 11494; Matthew (1906)

*S. lania*: AMNH FM 13142

*S. major*: USNM 540694

*S. minor*: AMNH FM 11532

*S. rapax*: USNM 13305, 13306, 13307, 13309, YPM-PU 10244, YMP 12862

*Tritemnodon agilis*: AMNH FM 11536, USNM 11544, 361351, YPM 10073, 11877; Matthew (1906, 1909)

*Prototomus martis*: UM 67138, USNM 509700, 511037, 511057, 511087, 511088, 511089, 511090, 525550, 527677

*Pyrocyon dioctetus*: UM 94757

*Pyrocyon strenuus*: AMNH FM 4781, USGS 6111, 16474, 16475, 27236, USNM 491821

*Arfia gingerichi*: Smith and Smith (2001); Solé et al. (2013)

*Arfia shoshoniensis/opisthotoma*

*A. opisthotoma*: UM 63591; Gingerich and Deutsch (1989)

*A. shoshoniensis*: CM 39126, UM 69474, 77051, 87768, UW 9915, YPM 36932; Gingerich and Deutsch (1989)

*Prolimnocyon* spp.: Polly (1996)

*P. atavus*: DPC 5364; USGS 9330, 12784, USNM 510977

*P*. *eerius*: Gingerich (1989)

*P*. *haematus*: Gingerich and Deutsch (1989)

*Limnocyon* spp.

*L. potens*: CM 11439

*L. verus*: AMNH FM 12155, USNM 299722; Matthew (1909)

*Thinocyon* spp.: Polly (1996)

*T. medius*: AMNH FM 12154, 13082, YPM 12874, 12881; Morlo and Gunnell (2003)

*T. velox*: Morlo and Gunnell (2003)

*Propterodon morrisi*: AMNH FM 20128*,* 21553, 21555, PIN 3107-320; Matthew and Granger (1925); Lavrov (1996)

*Propterodon tongi*: IVPP V12612; Liu and Huang (2002)

*Hyaenodon* spp.: Polly (1996)

*H. crucians*: AMNH FM 647, F:AM 75565; Mellett (1977)

*H. mustelinus*: Mellett (1977)

*H. raineyi*: Gustafson (1986)

*H. venturae*: Stock (1933); Lavrov and Emry (1998)

REFERENCES

Andrews, C. W. 1906. Catalog of the Tertiary Vertebrata of the Fayum, Egypt. Taylor and Francis, London, 324 pp.

Bajpai, S., V. V. Kapur, and J. G. M. Thewissen. 2009. Creodont and condylarth from the Cambay Shale (early Eocene, ~55-54 ma), Vastan Lignite Mine, Gujarat, western India. Journal of the Palaeontological Society of India 54:103-109.

Barry, J. C. 1988. *Dissopsalis*, a middle and late Miocene proviverrine creodont (Mammalia) from Pakistan and Kenya. Journal of Vertebrate Paleontology 8:25-45.

Bonis, L. d., F. Solé, Y. Chaimanee, A. N. Soe, C. Sein, V. Lazzari, O. Chavasseau, and J.-J. Jaeger. 2018. New Hyaenodonta (Mammalia) from the middle Eocene of Myanmar. Comptes Rendus Palevol 17:357-365.

Borths, M. R., P. A. Holroyd, and E. R. Seiffert. 2016. Hyainailourine and teratodontine cranial material from the late Eocene of Egypt and the application of parsimony and Bayesian methods to the phylogeny and biogeography of Hyaenodonta (Placentalia, Mammalia). PeerJ 4:e2639; DOI 10.7717/peerj.2639.

Borths, M. R., and E. R. Seiffert. 2017. Craniodental and humeral morphology of a new species of *Masrasector* (Teratodontinae, Hyaenodonta, Placentalia) from the late Eocene of Egypt and locomotor diversity in hyaenodonts. PLOS One 12:e0173527.

Borths, M. R., and N. J. Stevens. 2019a. Taxonomic affinities of the enigmatic *Prionogale breviceps*, early Miocene, Kenya. Historical Biology 31:784-793.

Borths, M. R., and N. J. Stevens. 2019b. *Simbakubwa kutokaafrika*, gen. et sp. nov. (Hyainailourinae, Hyaenodonta, ‘Creodonta,’ Mammalia), a gigantic carnivore from the earliest Miocene of Kenya. Journal of Vertebrate Paleontology 39:e1570222 (20 pages).

Colbert, E. H. 1933. The skull of *Dissopsalis carnifex* Pilgrim, a Miocene creodont from India. American Museum Novitates 603:1-8.

Crochet, J.-Y. 1988. Le gisement du Bretou (Phosphorites du Quercy, Tarn-et-Garonne, France) et sa faune de vertébrés de l’Éocène supérieur. III. Marsupiaux, créodontes et fissipèdes. Palaeontographica Abteilung A 205:51-67.

Crochet, J.-Y. 1991. A propos de quelques Créodontes Proviverrinés de l'Eocène supérieur du Sud de la France. Neues Jahrbuch für Geologie und Paläontologie, Abhandlungen 182:99-115.

Crochet, J.-Y., F. Crouzel, and B. Lange-Badré. 1976. Conséquences de la découverte du genre *Oxyaenoïdes* Matthes, sur la datation du Poudingue de Palassou. Comptes Rendus de l'Académie des Sciences, Serie D 282:1597-1600.

Crochet, J.-Y., H. Thomas, J. Roger, S. Sen, and Z. Al-Sulaimani. 1990. Première découverte d'un créodonte dans la péninsule Arabique: *Masrasector ligabuei* nov. sp. (Oligocène inférieur de Taqah, Formation d'Ashawq, Sultanat d'Oman). Comptes Rendus de l'Académie des Sciences, Paris, Série II 311:1455-1460.

Egi, N., P. A. Holroyd, T. Tsubamoto, A. N. Soe, M. Takai, and R. L. Ciochon. 2005. Proviverrine hyaenodontids from the Eocene of Myanmar and a phylogenetic analysis of the proviverrines from the Para-Tethys area. Journal of Systematic Palaeontology 3:337-358.

Gingerich, P. D. 1989. New earliest Wasatchian mammalian fauna from the Eocene of northwestern Wyoming: composition and diversity in a rarely sampled high-floodplain assemblage. University of Michigan Papers on Paleontology 28:1-97.

Gingerich, P. D., and H. A. Deutsch. 1989. Systematics and evolution of early Eocene Hyaenodontidae (Mammalia, Creodonta) in the Clarks Fork Basin, Wyoming. Contributions from the Museum of Paleontology, The University of Michigan 27:327-391.

Ginsburg, L. 1980. *Hyainailouros sulzeri*, mammifère créodonte du Miocène d'Europe. Annales de Paléontologie 66:19-73.

Gustafson, E. P. 1986. Carnivorous mammals of the late Eocene and early Oligocene of Trans-Pecos Texas. Texas Memorial Museum Bulletin 33:1-66.

Holroyd, P. A. 1994. An examination of dispersal origins for Fayum Mammalia. Ph.D. thesis/dissertation, Biological Anthropology and Anatomy, Duke University, Durham, NC, 328 pp.

Holroyd, P. A. 1999. New Pterodontinae (Creodonta: Hyaenodontidae) from the late Eocene-early Oligocene Jebel Qatrani Formation, Fayum province, Egypt. PaleoBios 19:1-18.

Kumar, K. 1992. *Paratritemnodon indicus* (Creodonta: Mammalia) from the early Middle Eocene Subathu Formation, NW Himalaya, India, and the Kalakot mammalian community structure. Paläontologische Zeitschrift 66:387-403.

Lange-Badré, B. 1979. Les créodontes (Mammalia) d'Europe occidentale de l'Éocéne supérieur a l'Oligocéne supérieur. Mémoires du Muséum National d'Histoire Naturelle, Série C 42:1-252.

Lange-Badré, B., and H. Haubold. 1990. Les Créodontes (Mammifères) du gisement du Geiseltal (Eocène moyen, RDA). Geobios 23:607-637.

Lange-Badré, B., and C. Mathis. 1992. Données nouvelles sur les Créodontes Proviverrinés des zones MP 16 et MP 17 des Phosphorites du Quercy. Bulletin du Museum National d'Histoire Naturelle, Section C 14:161-184.

Lavrov, A. V. 1996. A new genus *Neoparapterodon* (Creodonta, Hyaenodontidae) from the Khaichin-Ula-2 locality (Khaichin Formation, middle-upper Eocene, Mongolia) and the systematic position of the Asiatic *Pterodon* representatives. Paleontological Journal 30:593-604.

Lavrov, A. V., and R. J. Emry. 1998. *Hyaenodon venturae* (Hyaenodontidae, Creodonta, Mammalia) from the early Chadronian (latest Eocene) of Wyoming. Journal of Paleontology 72:752-757.

Lillegraven, J. A. 1969. Latest Cretaceous mammals of upper part of Edmonton Formation of Alberta, Canada, and review of marsupial-placental dichotomy in mammalian evolution. The University of Kansas Paleontological Contributions 50:1-122.

Liu, L., and X.-S. Huang. 2002. *Propterodon* (Hyaenodontidae, Creodonta, Mammalia) from the middle Eocene of Yuanqu Basin, Shanxi, China. Vertebrata PalAsiatica 40:133-138.

Matthew, W. D. 1906. The osteology of *Sinopa*, a creodont mammal of the middle Eocene. Proceedings of the United States National Museum 30:203-233.

Matthew, W. D. 1909. The Carnivora and Insectivora of the Bridger Basin, Middle Eocene. Memoirs of the American Museum of Natural History 9:291-567.

Matthew, W. D., and W. Granger. 1925. New mammals from the Irdin Manha Eocene of Mongolia. American Museum Novitates 198:1-10.

Mellett, J. S. 1977. Paleobiology of North American *Hyaenodon* (Mammalia, Creodonta). Contributions to Vertebrate Evolution 1:1-134.

Morales, J., M. Pickford, D. Soria, and S. Fraile. 1998. New carnivores from the basal middle Miocene of Arrisdrift, Namibia. Eclogae Geologicae Helvetiae 91:27-40.

Morlo, M., and G. F. Gunnell. 2003. Small limnocyonines (Hyaenodontidae, Mammalia) from the Bridgerian middle Eocene of Wyoming: *Thinocyon*, *Prolimnocyon*, and *Iridodon*, new genus. Contributions from the Museum of Paleontology, The University of Michigan 31:43-78.

Morlo, M., and J. Habersetzer. 1999. The Hyaenodontidae (Creodonta, Mammalia) from the lower middle Eocene (MP 11) of Messel (Germany) with special remarks to new x-ray methods. Courier Forschungsinstitut Senckenberg 216:31-73.

Osborn, H. F. 1909. New carnivorous mammals from the Fayûm Oligocene, Egypt. Bulletin of the American Museum of Natural History 26:415-424.

Peigné, S., M. Morlo, Y. Chaimanee, S. Ducrocq, S. T. Tun, and J.-J. Jaeger. 2007. New discoveries of hyaenodontids (Creodonta, Mammalia) from the Pondaung Formation, middle Eocene, Myanmar -- paleobiogeographic implications. Geodiversitas 29:441-458.

Polly, P. D. 1993. Hyaenodontidae (Creodonta, Mammalia) and the position of systematics in evolutionary biology. Ph.D. thesis/dissertation, University of California, Berkeley, Berkeley, 283 pp.

Polly, P. D. 1996. The skeleton of *Gazinocyon vulpeculus* gen. et comb. nov. and the cladistic relationships of Hyaenodontidae (Eutheria, Mammalia). Journal of Vertebrate Paleontology 16:303-319.

Rana, R. S., K. Kumar, S. P. Zack, F. Solé, K. D. Rose, P. Missiaen, L. Singh, A. Sahni, and T. Smith. 2015. Craniodental and postcranial morphology of *Indohyaenodon raoi* from the early Eocene of India, and its implications for ecology, phylogeny, and biogeography of hyaenodontid mammals. Journal of Vertebrate Paleontology 35:e965308 (22 pages).

Ranga Rao, A. 1973. Notices of two new mammals from the Upper Eocene Kalakot beds, India. Oil and Natural Gas Commission, special paper 2:1-6.

Rasmussen, D. T., C. D. Tilden, and E. L. Simons. 1989. New specimens of the giant creodont *Megistotherium* (Hyaenodontidae) from Moghara, Egypt. Journal of Mammalogy 70:442-447.

Savage, R. J. G. 1965. Fossil mammals of Africa: 19. The Miocene Carnivora of East Africa. Bulletin of the British Museum (Natural History), Geology series 10:242-316.

Savage, R. J. G. 1973. *Megistotherium*, gigantic hyaenodont from Miocene of Gebel Zelten, Libya. Bulletin of the British Museum (Natural History), Geology series 22:483-511.

Smith, T., and R. Smith. 2001. The creodonts (Mammalia, Ferae) from the Paleocene-Eocene transition in Belgium (Tienen Formation, MP7). Belgian Journal of Zoology 131:117-135.

Solé, F. 2013. New proviverrine genus from the early Eocene of Europe and the first phylogeny of late Palaeocene–middle Eocene hyaenodontidans (Mammalia). Journal of Systematic Palaeontology 11:375-398.

Solé, F., E. M. Essid, W. Marzougui, R. Temani, H. K. Ammar, M. Mahboubi, L. Marivaux, M. Vianey-Liaud, and R. Tabuce. 2016. New fossils of Hyaenodonta (Mammalia) from the Eocene localities of Chambi (Tunisia) and Bir el Ater (Algeria), and the evolution of the earliest African hyaenodonts. Palaeontologia Electronica 19.2.38A:1-23.

Solé, F., J. Falconnet, and D. Vidalenc. 2015. New fossil Hyaenodonta (Mammalia, Placentalia) from the Ypresian and Lutetian of France and the evolution of the Proviverrinae in southern Europe. Palaeontology 58:1049-1072.

Solé, F., J. Falconnet, and L. Yves. 2014a. New proviverrines (Hyaenodontida) from the early Eocene of Europe; phylogeny and ecological evolution of the Proviverrinae. Zoological Journal of the Linnean Society 171:878-917.

Solé, F., E. Gheerbrant, and M. Godinot. 2013. Sinopaninae and Arfianinae (Hyaenodontida, Mammalia) from the Early Eocene of Europe and Asia; evidence for dispersal in Laurasia around the Paleocene/Eocene boundary and for an unnoticed faunal turnover in Europe. Geobios 46:313-327.

Solé, F., J. Lhuillier, M. Adaci, M. Bensalah, M. Mahboubi, and R. Tabuce. 2014b. The hyaenodontidans from the Gour Lazib area (?early Eocene, Algeria): implications concerning the systematics and the origin of the Hyainailourinae and Teratodontinae. Journal of Systematic Palaeontology 12:303-322.

Stock, C. 1933. Hyænodontidæ of the upper Eocene of California. Proceedings of the National Academy of Sciences 19:434-440.

Szalay, F. S. 1967. The affinities of *Apterodon* (Mammalia, Deltatheridia, Hyaenodontidae). American Museum Novitates 2293:1-17.

Van Valen, L. M. 1965. Some European Proviverrini (Mammalia, Deltatheridia). Palaeontology 8:638-665.

Werdelin, L. 2019. Middle Miocene Carnivora and Hyaenodonta from Fort Ternan, western Kenya. Geodiversitas 41:267-283.

Wible, J. R., G. W. Rougier, M. J. Novacek, and R. J. Asher. 2009. The eutherian mammal *Maelestes gobiensis* from the Late Cretaceous of Mongolia and the phylogeny of Cretaceous Eutheria. Bulletin of the American Museum of Natural History 327:1-123.
